# Editorial: Unsuccessful Psychotherapies: When and How Do Treatments Fail?

**DOI:** 10.3389/fpsyg.2020.578997

**Published:** 2020-12-11

**Authors:** Osmano Oasi, Andrzej Werbart

**Affiliations:** ^1^Department of Psychology, Catholic University of the Sacred Heart, Milan, Italy; ^2^Department of Psychology, Stockholm University, Stockholm, Sweden

**Keywords:** unsuccessful psychotherapies, impasse, negative effects, dropout, variables implicated

As humans, but also as researchers and clinicians, we can learn a lot from our failures. The issue of failures in psychotherapeutic treatments is extremely important, as from them we can infer the signs that precede them, and the strategies to deal with them. In psychotherapy we are also aware of the important fact that the amount of unwanted effects is very similar to fields such as pharmacotherapy, and the number of patients reporting unwanted effects of psychotherapy is between 3 and 15% of cases (Berk and Parker, 2009). In 2012, a meta-analytic study by Swift and Greenberg (2012) suggested that approximately one in every five clients still chooses to end treatment prior to its completion. Similarly, Lambert (2013) has demonstrated that 5 to 10% of patients deteriorate in therapy, and 35 to 40% of participants in clinical trials do not improve. This Research Topic asks how we can address this situation.

Technical mistakes by psychotherapists or the particular mental conditions of patients are typical variables that lead to unsuccessful and negative outcomes. In early pioneering clinical studies, both variables can be observed, for example, the transference/countertransference phenomena in Breuer's case of Anna O and Dora's drop out from treatment with Freud. Subsequently, several difficulties have arisen in the study of unsuccessful psychotherapies: (1) the methodological proposals for studying positive effects often obscure negative effects; (2) the complexity of the therapeutic process; and, (3) a lack of agreement on the definition of treatment failures. Indeed, treatment failure has been used as an umbrella term for a broad array of unwished-for effects of psychotherapy, such as attrition, lack of change, relapse, and a worsening of patient conditions. Additional challenges when measuring outcome and defining therapeutic success and failure include: which perspective is being used (patient, therapist, or researcher), what types of outcomes are measured with which methods, and the appropriate time point of outcome monitoring.

Over the last decade, psychotherapy was considered to be a complex form of interaction, which is in many ways different from relationships in ordinary life. Regardless of the specific therapeutic method, the role of the therapist is to facilitate change in a patient and to improve their functioning. From the researcher's perspective, treatment failures have been related to negative interpersonal processes in psychotherapy. Furthermore, unrepaired ruptures are connected to the unilateral interruption and drop out of patients (Safran et al., 2011; Gülüm et al., 2018; Colli et al., 2019). There is significant evidence of substantial variance in treatment outcomes between different therapists. Therapists may be more important for therapeutic success than the type of intervention they deliver. Furthermore, while therapists differ in their average outcomes, most therapists have at least some successful case outcomes. On the other hand, even the most effective therapists have experienced unsuccessful treatments when patients did not improve.

The articles presented in this Research Topic show how complex the topic is. In an evidence-based case study, Block-Elkouby et al. suggest the combination of at least three groups of factors. A first group refers to factors related to the patient, a second to those related to the psychotherapist, and a third to those attributable to the expected treatment modality. A tentative process model of the development of suboptimal psychotherapy with young adults, presented by von Below, focuses on when therapists underestimate patient problems, patient pseudo-mentalizing, and no development toward agency. Adopting another perspective, Carcione et al. investigate the role of mentalization in the process of change and demonstrate that changes in metacognition predict improvements in personality disorders. The work of Braga et al. shows that an analytic procedure based on coding ambivalence can predict subsequent symptomatology, thus helping therapists to promote moments of communication between the opposing positions of the patient's self.

Several of the studies included in this Research Topic address different aspects of dropout. Maggio et al. study cases of premature termination and hypothesize that the particular emotional responses of the therapist may be prognostic. O'Keeffe et al. investigate types of dropout from the perspective of adolescent clients: “dissatisfied” dropouts experienced the therapy as not helpful, “got-what-they-needed” dropouts experienced sufficient benefits from therapy, whereas “troubled” dropouts lacked stability in their lives. Smith et al. conclude that war veterans with more PTSD symptomatology and those who did not receive trauma-focused therapy were less likely to complete residential treatment. A meta-analysis of non-response in internet-based CBT conducted by Rozental et al. focuses on patient variables and found that higher symptom severity and male gender increase the odds of a patient not responding.

In a grounded theory study, De Smet et al. found that non-improved depressed patients experienced a stalemate in therapy, stuck between knowing and doing. Nevertheless, “no change” in outcome scores involved, from the patients' perspective, a “partial change.” Werbart et al. studied contrasting cases from the caseloads of three therapists. In successful treatments, the patients and the therapists shared a joint view of the therapy and their relationship, whereas in unsuccessful treatments their views diverged and the therapists had difficulty in reflecting on their contributions. Curran et al. undertook a meta-synthesis of service user experiences, revealing potentially harmful factors at each stage of the therapy process that may require adequate remedial action.

Taken together, the 11 articles included in this Research Topic demonstrate that researchers and clinicians can learn a great deal from further studies of unsuccessful treatments. To do so, we need to look not only for patient factors, but also the therapist contributions, as well as the therapeutic relationship. As proposed by the third APA task force, we have to “identify effective elements of the therapy relationship and to determine effective methods of adapting or tailoring therapy to the individual patient on the basis of transdiagnostic characteristics” (Norcross and Wampold, 2019, p. 3). The therapist's ability to recognize and manage ruptures is decisive in the repair process and the prevention of treatment failure (Eubanks-Carter et al., 2011). A focus on the emotional reactions of the therapist determined by their specific personality traits, for example, narcissism (Oasi et al., 2019), is of fundamental importance. A further crucial issue is a patient-therapist match in terms of personality orientation, and an early adjustment by the therapist, in terms of their orientation on relatedness or self-definition, to the patients' predominant personality configuration, might enhance treatment outcomes (Werbart et al., 2018). Even the well-known construct of therapeutic alliance can receive (and give) further strength if it is placed in a relational context. Taking up the groundbreaking statement by Horwitz et al. (1996), we run fewer risks if we are able to tailor our way of working to the patient.

## Author Contributions

All authors listed have made a substantial, direct and intellectual contribution to the work, and approved it for publication.

## Conflict of Interest

The authors declare that the research was conducted in the absence of any commercial or financial relationships that could be construed as a potential conflict of interest.
